# Modified behavioural tests to detect white matter injury- induced motor deficits after intracerebral haemorrhage in mice

**DOI:** 10.1038/s41598-019-53263-6

**Published:** 2019-11-18

**Authors:** Weixiang Chen, Min Xia, Chao Guo, Zhengcai Jia, Jie Wang, Chengcheng Li, Mingxi Li, Xiaoqin Tang, Rong Hu, Yujie Chen, Xin Liu, Hua Feng

**Affiliations:** 1 Department of Neurosurgery, Southwest Hospital, Third Military Medical University (Army Medical University), 29 Gaotanyan Street, Shapingba District, Chongqing 400038 China; 2 Department of Neurosurgery, Southwest Hospital, Collaborative Innovation Center for Brain Research, Third Military Medical University (Army Medical University), 29 Gaotanyan Street, Shapingba District, Chongqing 400038 China

**Keywords:** Stroke, White matter injury

## Abstract

Motor function deficit induced by white matter injury (WMI) is one of the most severe complications of intracerebral haemorrhage (ICH). The degree of WMI is closely related to the prognosis of patients after ICH. However, the current behavioural assessment of motor function used in the ICH mouse model is mainly based on that for ischaemic stroke and lacks the behavioural methods that accurately respond to WMI. Here, a series of easy-to-implement behavioural tests were performed to detect motor deficits in mice after ICH. The results showed that the grip strength test and the modified pole test not only can better distinguish the degree of motor dysfunction between different volumes of blood ICH models than the Basso Mouse Scale and the beam walking test but can also accurately reflect the severity of WMI characterized by demyelination, axonal swelling and the latency of motor-evoked potential delay induced by ICH. In addition, after ICH, the results of grip tests and modified pole tests, rather than the Basso Mouse Scale and the beam walking test, were worse than those observed after intraventricular haemorrhage (IVH), which was used as a model of brain haemorrhage in non-white matter areas. These results indicate that the grip strength test and the modified pole test have advantages in detecting the degree of motor deficit induced by white matter injury after ICH in mice.

## Introduction

Intracerebral haemorrhage (ICH) has a high incidence (120/100000) and mortality (2/3 of the survivors) worldwide^1^. Motor deficit induced by white matter injury (WMI) is one of the most severe complications that impairs quality of life and can be used as a predictor of prognosis in ICH patients^2,3^. ICH occurs most frequently in the basal ganglia and damages the local white matter conduction tracts, mainly the corticospinal tract^4,5^. After basal ganglion haemorrhage, patients will have obvious hemiplegic symptoms due to a reduction in contralateral muscle strength and motor dysfunction is the main prognostic indicator of patients^6,7^. In patients, the muscle strength of the contralateral limb can be detected directly with instructions. However, most of the existing methods for evaluating intracerebral haemorrhage in mice are directly derived from cerebral ischemia, and lack of assessment of motor dysfunction caused by white matter destruction in basal ganglia. Thus, appropriate behavioural methods are urgently needed to address the WMI-induced motor deficits after basal ganglion haemorrhage^8,9^.

To better evaluate the motor dysfunction of white matter injury after intracerebral haemorrhage, six simple behavioural methods were performed. We either (1) improved a previous test (the modified pole test)^10^ (2) investigated whether existing tests (Grip strength tests and Bosso Mouse Scale)^11,12^ are viable to assess motor deficits after ICH or (3) evaluated existing post-ICH motor deficit tests (corner turn test and beam walking test)^13,14^. The latency of MEPs was used to detect injury to the corticospinal tract (CST), which may reflect white matter injury around the haematoma and it can predict motor function recovery after ICH^15–17^. The intraventricular haemorrhage (IVH) model was selected as a non-white-matter-located brain injury model. Different volumes of blood injection models were also used to further evaluate the behavioural methods^2^.

Based on our research, the grip strength tests and the modified pole test correlated with the histological examination of the white matter and the change in MEPs. These tests could better evaluate the degree of motor deficits between IVH and ICH as well as different blood volumes in the ICH mouse model. The grip strength test and the modified pole test should be widely used when assessing the extent of white matter injury and the role of neuroprotective agents after ICH.

## Materials and Methods

### ICH and IVH models

All experimental protocols were approved by the Ethics Committee of the Third Military Medical University (Army Medical University) and performed according to the health guide for the care and use of laboratory animals. Healthy male C57BL/6 N mice weighing 23–26 g were purchased from the Experimental Animal Center at the Third Military Medical University (Army Medical University, permit number: Yu2017-0002; Chongqing, China) at 7 weeks of age. Animals were randomly divided into different experimental groups.

Animals were anaesthetized with halothane (70% N_2_O and 30% O_2_; 4% induction, 2% maintenance) and then fixed on a stereotactic instrument (RWD Life Sciences Ltd.), and 10 µl or 25 µl of autologous blood was injected into the right caudate nucleus. The needle used here is a 25 µl Neuros syringe (Needle inner diameter: 0.108 mm; Needle outer diameter: 0.210 mm; Needle length: 0–20 mm adjustable: 65460-10, Hamilton). The position relative to the right caudate nucleus and anterior iliac crest was 0.8 mm in front, 2.5 mm beside, and 3.0 mm in depth, as previously described^18^. Twenty-five microliters of normal saline was injected into the saline group, and an operation procedure without compound injection was performed in the sham operation group. The skull was sealed with bone wax, and a suture was applied to the scalp to complete the craniotomy. Body temperature was maintained at 37 ± 0.5 °C throughout the experiment and recovery period from anesthesia (about 1 hour). For the IVH model, the position was 1.0 mm in front, 1.5 mm beside, and 2.5 mm in front, and 25 µl of blood was injected^19^. The other steps were the same as those for the ICH model, and sham-operated mice were subjected only to needle insertion.

### Immunohistochemistry

The brain was removed by fixed infusion with 4% paraformaldehyde and then immersed in 30% sucrose in phosphate buffered saline (PBS). Serial sections were cut on a freezing microtome, blocked, and incubated in the following primary antibodies: rabbit anti-Iba1 (diluted 1:500, Wako, 019-19741), goat anti-GFAP (diluted 1:500, Santa Cruz, sc-6171), goat anti-MBP (diluted 1:500, Santa Cruz, sc-13914), and rabbit anti-neurofilament 200 (NF200) (1:200; Sigma-Aldrich; Cat.: N4142). After washing, the sections were incubated with the appropriate fluorescent secondary antibody (AlexaFluor-488- and AlexaFluor-555-conjugated antibodies (diluted 1:1000, Invitrogen)) and counterstained with DAPI. Images of the surrounding haematoma in each section were captured by a Zeiss microscope (Zeiss, LSM780, Germany). The microscopic field around the haematoma was analysed in three brain slices randomly selected under the blinded principle.

### Transmission electron microscopy

For transmission electron microscopy (TEM), animals were perfused with 1.25% glutaraldehyde and 2% paraformaldehyde in 0.1 M PB after initial irrigation with isotonic saline. Then, the brain was rapidly removed and fixed at 4 °C for 4 days. The tissue was washed and fixed with 1% OsO_4_ in PB for 2 hours, counterstained with uranyl acetate, dehydrated with a graded acetone series, infiltrated with propylene oxide, and embedded in Epon. Ultrathin sections (~60 nm) were cut by an ultramicrotome (LKB-V, LKB Produkter AB, Bromma) and observed under a transmission electron microscope (TECNAI10; Philips)^20^. Random images of 12 different fields of view were selected for each animal for the statistical analysis of myelinated axons. The G-ratio of myelinated fibres was calculated as the ratio of the diameter of the axon to the diameter of the axon with the myelin sheath by using ImageJ software (ImageJ 1.8; NIH, Bethesda, MD), and at least 60 myelinated fibres in each animal were calculated^21^.

### Behaviour tests

#### Modified pole test

The frontlimbs of the mice were placed on a pole with a horizontally rough surface (diameter 8 mm). Each test lasted 30 seconds and was repeated three consecutive times. The scores are representative of when the blood injection position was on the right side of the brain. Scoring proceeded as follows: 1 point, the mouse grabbed the bottom of the horizontal bar for 1–10 seconds and then dropped; 2 points, the mouse grabbed the bottom of the horizontal bar, held on for 11–20 seconds and then dropped; 3 points, the mouse grabbed the bottom of the horizontal bar, held on for 21–30 seconds and then dropped; 4 points, the mouse grabbed the bottom of the horizontal bar for 30 seconds, but the left hindlimb and left forelimb were hanging down the pole; 5 points, the mouse grabbed the bottom of the horizontal bar for 30 seconds, but the left hindlimb or left forelimb was hanging down the pole; 6 points, the mouse firmly grasped the bottom of the horizontal bar pole for 30 seconds but could not sit on it; 7 points, the mouse sat on the pole, but the left hindlimb and left forelimb were hanging down; 8 points, the mouse sat on the pole, but the left hindlimb or forelimb was hanging down; and 9 points, the mouse moved freely on the pole and all limbs were stable.

#### Beam walking test

The mouse was allowed to cross a round wooden beam 1.5 cm in diameter and 70 cm in length elevated 30 cm from the ground. After the mouse crossed, the corresponding scores were obtained. The test was repeated three times, and the score (0–4 points) was determined by walking distance and gait. The average score of three consecutive trials was calculated. Higher scores indicated a better test performance: 0 points, the mouse could not grasp the wooden beam or sit on the wooden beam and fell directly; 1 point, the mouse could grasp the wooden beam or sit on the wooden beam, could not move, but could stay on for 1 minute; 2 points, the mouse maintained balance on the wooden beam, could not cross the beam, but could stay on for 1 minute; 3 points, the mouse could walk from one end of the beam to the other, but footfall occurred; and 4 points, the mouse could freely move from one end of the beam to the other end of the beam^22^.

#### Basso mouse scale (BMS)

This open-field locomotor scoring system ranges from 0 to 9 points: 0 points, no ankle movement; 1 point, slight ankle movement; 2 points, extensive ankle movement; 3 points, plantar placement with or without weight support; 4 points, occasional plantar stepping; 5 points, frequent or consistent plantar stepping, no coordination; 6 points, frequent or consistent plantar stepping, some coordination, paws parallel at initial contact; 7 points, frequent or consistent plantar stepping, mostly coordinated, paws parallel at initial contact and rotated at lift off; 8 points, frequent or consistent plantar stepping, mostly coordinated, paws parallel at initial contact and lift off, and mild trunk instability; and 9 points, frequent or consistent plantar stepping, mostly coordinated, paws parallel at initial contact and lift off, and normal trunk stability and tail always up^7,12^.

#### Corner turn test

The mouse was allowed to enter a corner with an angle of 30 degrees. To leave the corner, the mouse could turn either to the left or the right, and this was recorded. This test was repeated 10 times, with at least 30 seconds between trials, and the percentage of right turns was calculated^23^.

#### Grip strength test

The grip strength of the hindlimbs and all limbs was measured by a grip strength metre (Laboratory Enterprises, Nasik, India), which consisted of a steel wire grid (8 × 8 cm) connected to an isometric force transducer following the method described earlier^24^, and was recorded as the grip strength of the hindlimb and the grip strength of all limbs, respectively. Each mouse was measured in triplicate, and the average handgrip strength of each mouse was recorded. Grip strength was measured with a computerized grip strength metre. The peak force of each measurement was recorded in grams (g). This value was considered 100% of the grip strength. The animal was lifted by its tail so that it could grasp the grid with its forepaws. The mouse was then gently pulled back until it released the grid, and the maximal force in newtons (N) exerted by the mouse before losing grip was measured. The procedure was repeated three times, and the mean force exerted by each mouse before losing its grip was recorded. The mean force was then normalized to body weight and is expressed in mN/g^25–27^.

All the behaviour tests were performed by a blinded investigator.

#### Electrophysiological assessment

To record the motor-evoked potentials (MEPs) elicited by transcranial electrical stimulation, 6 mice per group were anaesthetized with 1% pentobarbital sodium (25 mg/kg i.p.). Previous studies showed that this dose of pentobarbital sodium did not significantly affect the amplitude or waveform of MEPs in dogs and rodents^7,15^. Electrode placement was described in a previous study^15^. The stimulation needle electrodes were placed subcutaneously with the tip touching the scalp. The cathode was placed at the midpoint of an imaginary line connecting the two ears and the anode at the base of the nose. The hindlimbs of mice were exposed to enable the insertion of recording electrodes into the gastrocnemius muscles. A ground electrode was placed subcutaneously in the back. Electrical stimulation was applied to excite the brain using a stimulator (Keypoint, Medtronic, USA). A single pulse of stimulation (7.8 mA, 0.1 ms, 1 Hz) was delivered via stimulation needle electrodes (DSN1620, Medtronic, USA). Electrical stimulation was repeated five times at intervals of 15 seconds in each mouse, and a trace of one of the five stimuli is presented as a representative example in the figure. The trace represents the potential change that occurred after a single transcranial electrical stimulation, and two wave groups appeared after the stimuli. The second and longer latency MEPs after transcranial electrical stimulation reflected the transduction of motor corticospinal tract^7^. The long latency of the MEPs was recorded for analysis and was measured from the onset of the transcranial electrical stimulation to the beginning of the evoked event. The average long latency of these 5 stimuli was used for statistics^17,28^.

### Statistical analysis

Values are given as the mean ± S.D. SPSS 11.5 (SPSS, Inc., Chicago, IL, USA) was used for statistical analysis. The behavioural experiments which were done at multiple time points were applied by two-way ANOVA with repeated-measure. The correlation between the percent change in different behavioural tests and the latency of the MEPs (C-E) was analysed by Pearson correlation coefficients. Other data were analysed by one-way ANOVA followed by the Scheffé F test for post hoc analysis or Student’s t-test. P < 0.05 was considered statistically significant.

## Results

### White matter damage and glial cell activation after ICH

**Figure 1:**
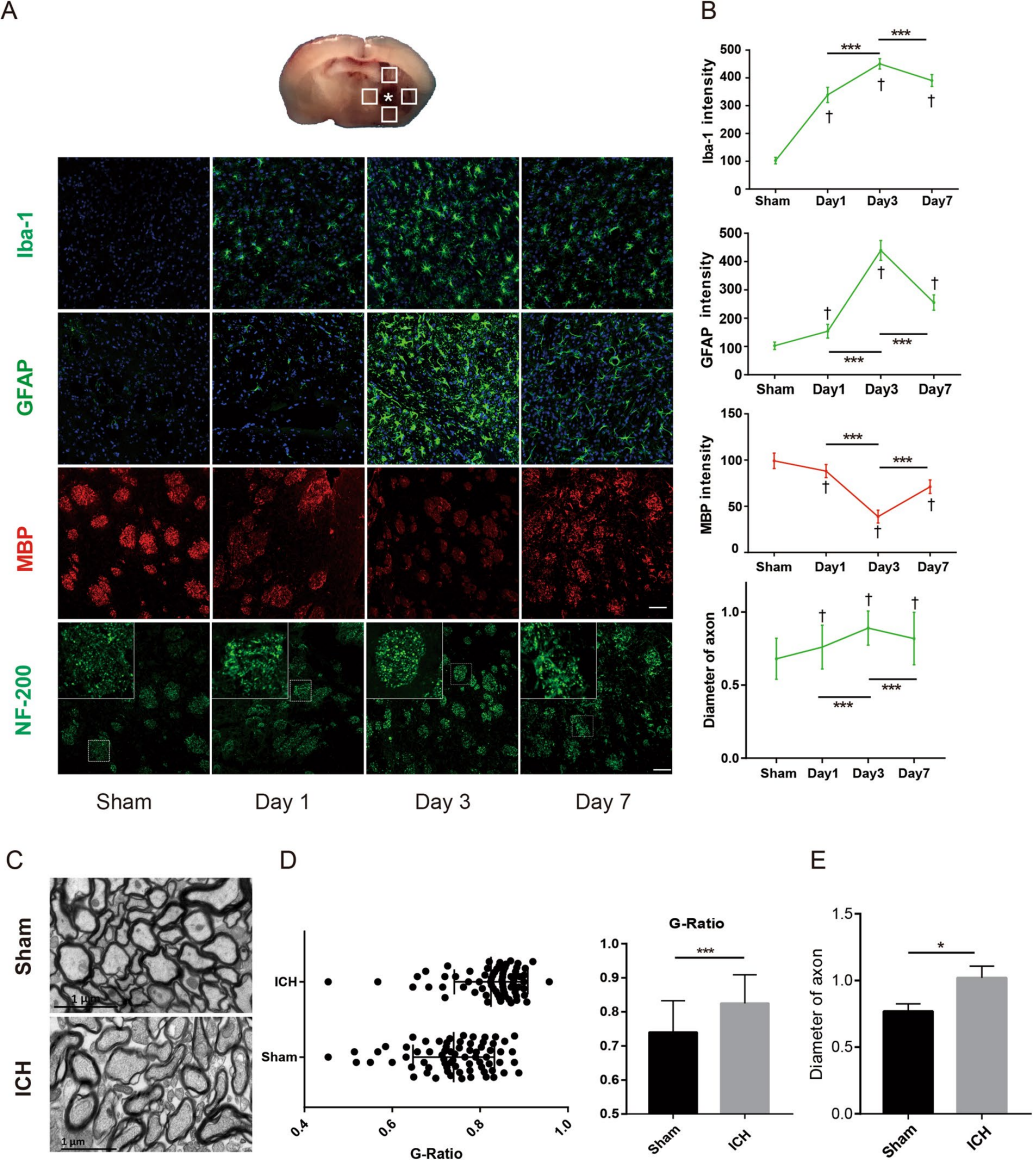
Glia cell activation and myelin damage were assessed from day 1 to day 7 after ICH. (**A**) The schematic diagrams show the four areas (white squares) for immunofluorescence photography in the perihaematomal region, and the white star indicates the haematoma. Iba1, GFAP-positive cells and MBP in brain sections were identified on days 1, 3 and 7 after ICH. Immunoreactivity of Iba1 (ionized calcium-binding adaptor molecule 1) and GFAP (glial fibrillary acidic protein; astrocyte marker) is shown in green, MBP (myelin basic protein) is shown in red, and NF-200 (neurofilament-200) is shown in green. Nuclei were stained with DAPI (blue). Scale bars: 50 μm (inset column). (**B**) Intensity of Iba-1, GFAP and MBP staining and diameter of NF-200 were quantified; n = 3 mice and 6 pictures per time point. (**C**) TEM shows the myelin sheath around the haematoma on day 3 after ICH. (**D**) G-ratio of different groups (n = 60, 3 mice). (**E**) Diameter of axons (n = 6 slices, 3 mice). Values are shown as the mean ± S.D., *P < 0.05, **P < 0.01, ***P < 0.001; ^†^P < 0.05 vs. sham.

Microglia and astrocytes are the major cell types that respond to the release of metabolites by haematomas after ICH, and the activation of microglia and astrocytes contributes to inflammation and swelling in white matter^29,30^. The activated microglia (Iba1 + cells) and astrocytes (GFAP + cells) were assembled in the internal capsule and were significantly increased before day 7 after ICH (Fig. 1A). In the sham group, glia cell and white matter shows no significant difference from day 1 to day 7. However, both Iba1 + and GFAP + signals were the highest on day 3 compared with those on day 1 and day 7 (P < 0.01), indicating that inflammation was the most serious at day 3 after ICH (Fig. 1B). MBP and NF200 were used to assess the white matter after ICH. The intensity of MBP was the lowest on day 3 compared with those on day 1 and day 7 (P < 0.001), indicating the most serious injury to myelin (Fig. 1A,B). The diameter of axons in the internal capsule around the haematoma was determined with neurofilament-200, and the diameter showed the most severe swelling on day 3 (0.89 ± 0.117 μm).

The ultrastructure of conduction tracts in the internal capsule was revealed by transmission electron microscopy, and tracts in 3 days after ICH showed a thinner myelin sheath and a swollen axon when compared with those of sham-operated mice (G-ratio; sham, 0.73 ± 0.09; ICH, 0.82 ± 0.08; diameter of the axons; sham, 0.76 ± 0.05 μm; ICH, 1.02 ± 0.09 μm).

### The behavioural tests were used to detect the motor deficits after ICH

**Figure 2:**
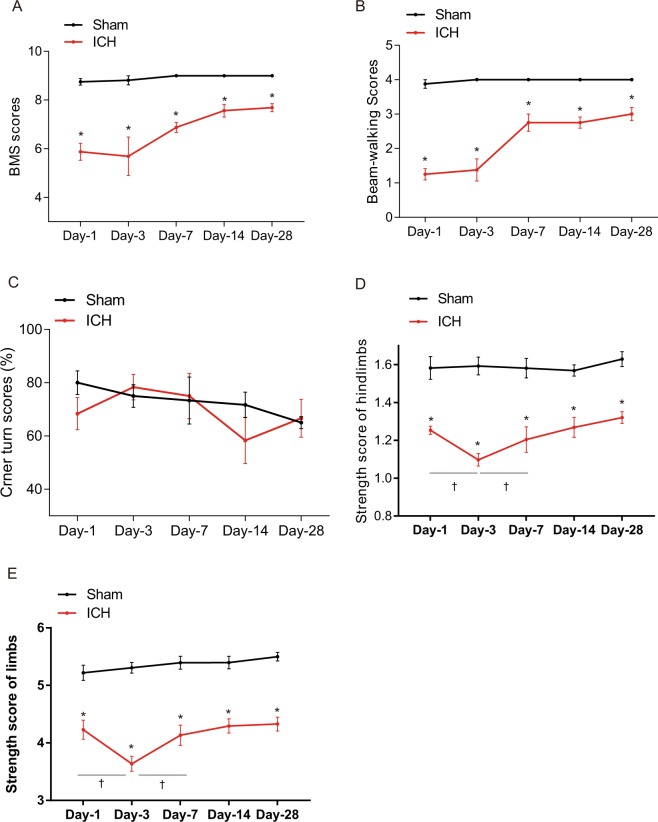
Behavioural tests were used to detect behaviour recovery after ICH. (**A**) BMS scores; (**B**) beam walking scores; and (**C**) corner turn test; data are expressed as the median and interquartile range. (**D**) Grip strength test of the hindlimbs and E: grip strength test of all limbs. Data represent the mean ± S.D., n = 9 for each group; *P < 0.05 and **P < 0.01, ^†^P < 0.05 vs. day 3.

Motor functions were detected by the BMS, beam walking test, corner turn test and grip strength test from day 1 to day 28 after ICH. The results showed that the BMS, beam walking test and grip strength test could distinguish the degree of motor deficits of mice after 25ul blood ICH model compare with the sham group from day 1 to day 28 (P < 0.05, Fig. 2A,B,D,E). In the corner turn test, the mice in the ICH and sham groups showed no significant differences on days 3, 7, 14, or 28 (Fig. 2C). The results of grip strength assessment showed that the worst muscle strength was on the third day.

### A modified pole test could detect hemiplegia in an ICH mouse model

**Figure 3:**
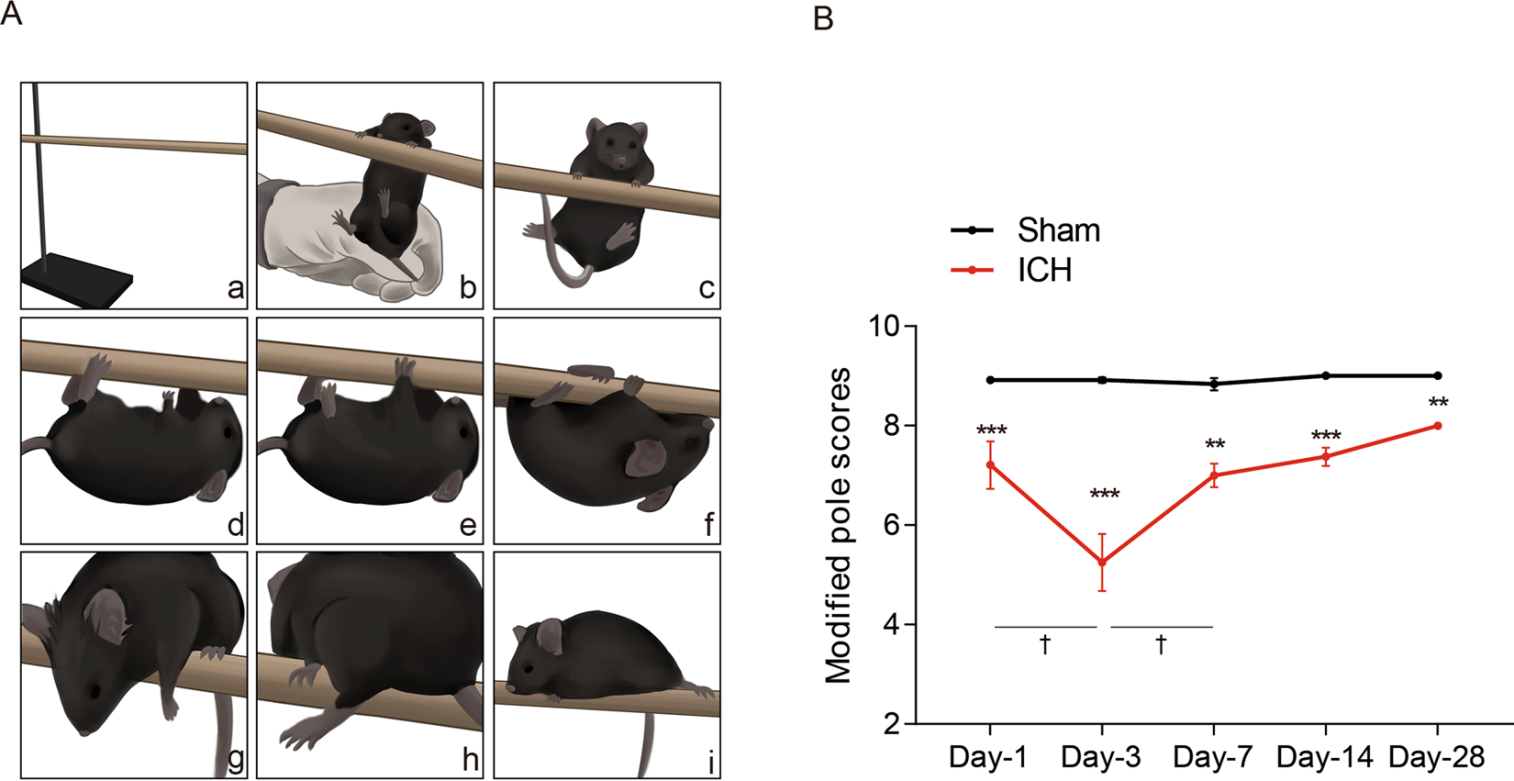
A modified pole test was used to detect hemiplegia after ICH. (**A**) The state of the mouse in the modified pole test. a: The equipment of pole; b and c: The initial state of the test; d: The mouse grabbed the bottom of the horizontal bar with left frontlimb hanging down; e: The mouse grabbed the bottom of the horizontal bar with left hindlimb hanging down; f: The mouse firmly grasped the bottom of the horizontal bar with all the limbs; g: The mouse stay on the pole with left frontlimb hanging down; h: The mouse stay on the pole with left hindlimb hanging down; i: The mouse stay on the pole with all limbs. (**B**) Modified pole test scores of mice after ICH. Values are shown as the mean ± S.D., n = 9 for each group, *P < 0.05, **P < 0.01, ***P < 0.001; ^†^P < 0.05 vs. day 3.

The detailed grading rules are described in the methods. Figure 3A shows the different postures of mice that were used to define their scores (details are described in the methods). The scores of ICH mice compared with sham mice significantly decreased from day 1 to day 28 (P < 0.05) (Fig. 3A,B). The results showed that the worst motor deficit was on the third day.

### The correlation between different behavioural tests and the latency of motor-evoked potentials

**Figure 4:**
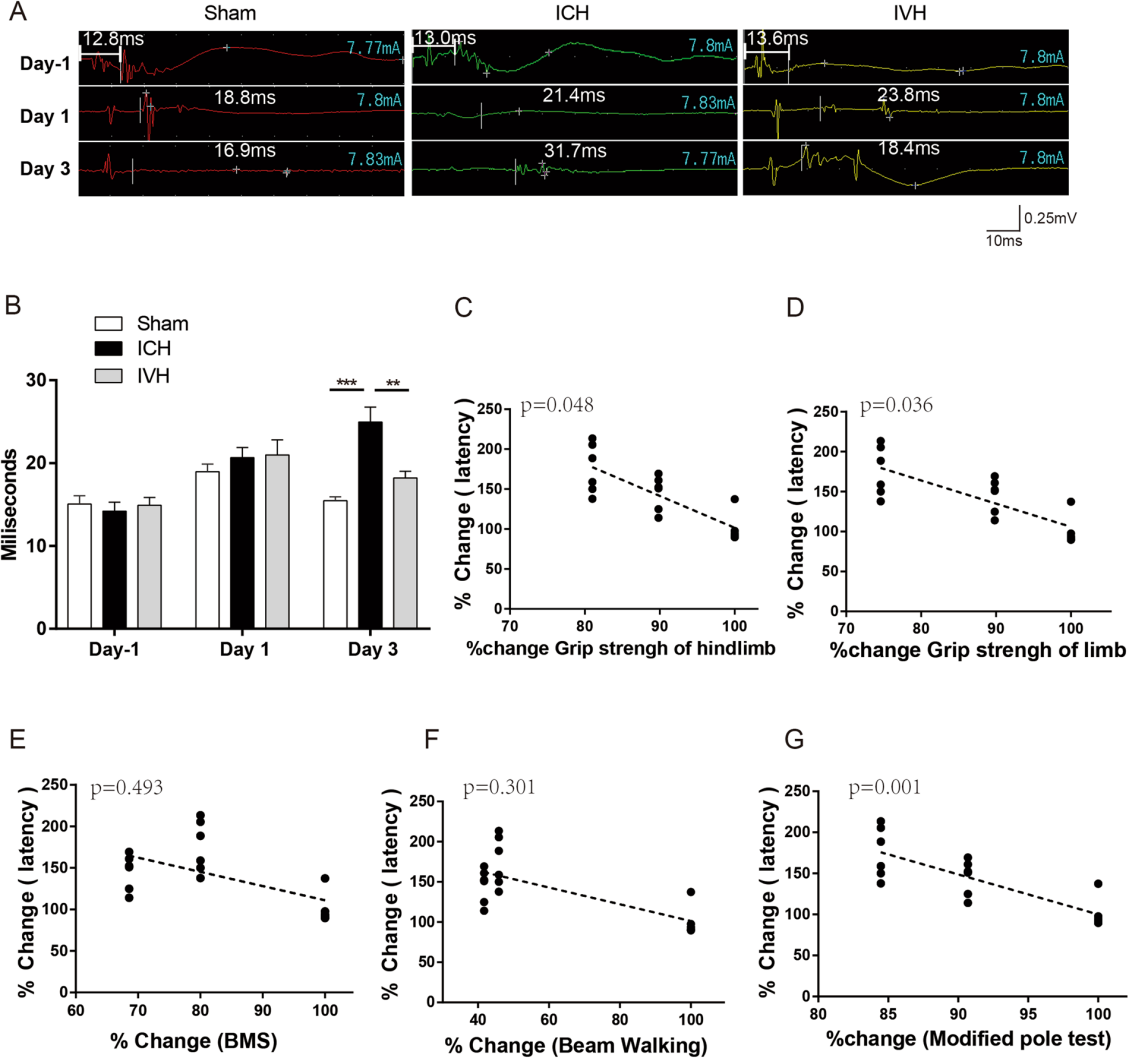
The latency of motor-evoked potentials (MEPs) was quantified after ICH and IVH (**A**,**B**). The white vertical lines represent the long latency of MEPs. The correlation between the percent change of different behavioural tests and the latency of MEPs (**C**–**E**), n = 6 mice per group. Values are expressed as the mean ± S.D., **P < 0.01, ***P < 0.001.

The latency of MEPs was used to detect the damage to the cortical spinal pathway that resulted from white matter injury in ICH/IVH (Fig. 4A). The latency was indicated in the Fig. 4A. And the latency of MEPs was 14.7 ± 0.53 ms before treatment (sham: 15.03 ± 0.53 ms, ICH: 14.2 ± 0.98 ms, IVH: 14.91 ± 0.85 ms). There was no significant difference between the three groups on day 1 after treatment (sham: 18.96 ± 0.83 ms, ICH: 20.65 ± 1.13 ms, IVH: 20.97 ± 1.69 ms) (Fig. 4B). However, on day 3 after intervention, the latency in the ICH group was significantly longer than that in the sham and IVH groups, and the latency increased at day 3 compared to day 1 but only in the ICH group (sham: 15.47 ± 0.44 ms, ICH: 24.95 ± 1.65 ms, IVH: 18.21±0.73 ms) (Fig. 4B).

The percent change in latency correlated with the percent change in the grip strength test and modified pole test but did not correlate with the percent change in the BMS and beam walking test (Fig. 4C–G). After day 3, the MEP latency began to shorten, and the neurological behavioural scores began to improve. From day 3 to day 14, the percent change in latency correlated with the percent change in the grip strength test and modified pole test but did not correlate with the percent change in the BMS and beam walking test (Supplementary Fig. 3).

### ICH mice behaved significantly worse than IVH mice in the strength test and modified pole test

**Figure 5:**
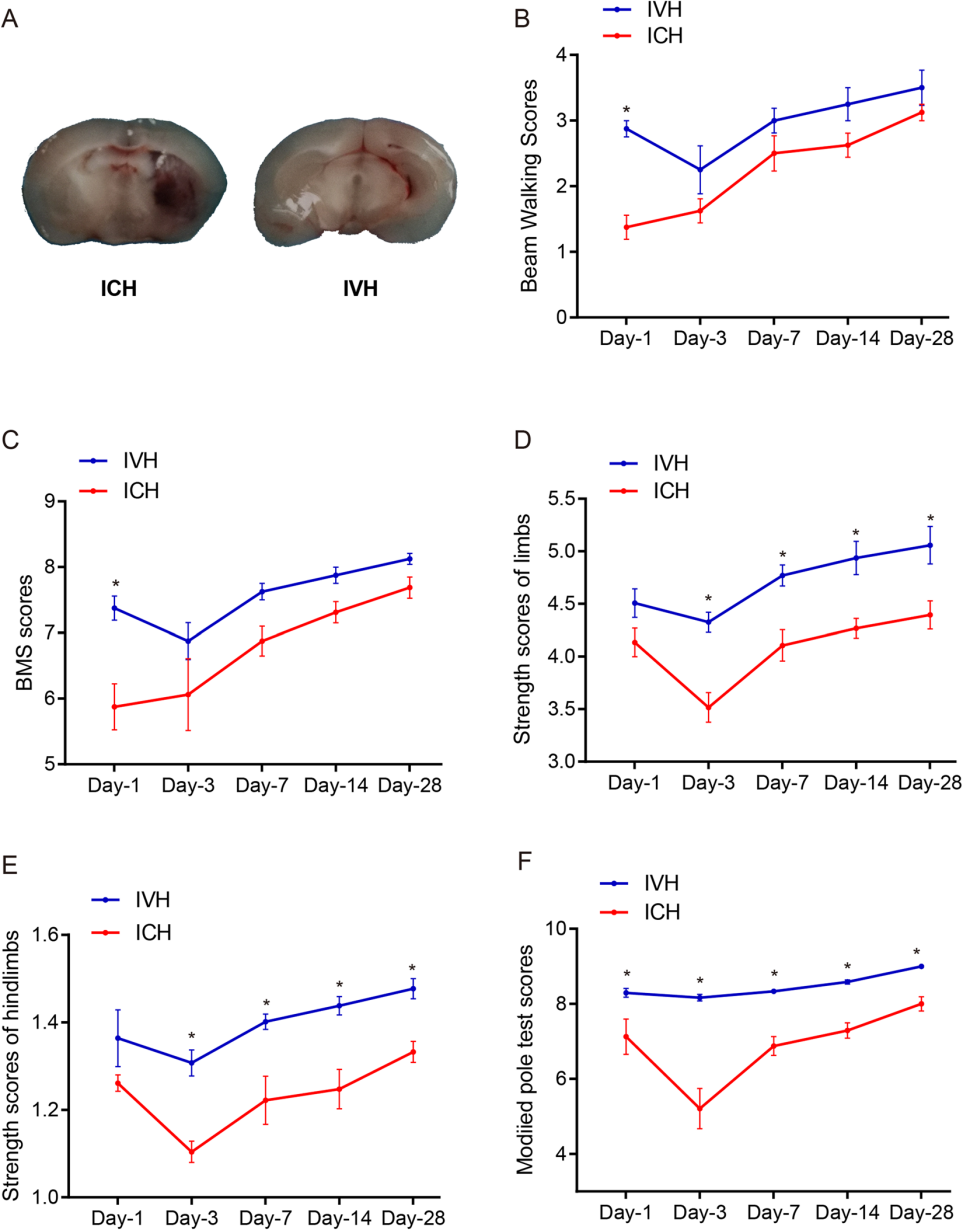
Mice with ICH and IVH were examined by different behavioural methods. (**A**) The haematoma after ICH or IVH at day 3. (**B**–**F**) Behavioural tests of mice after ICH and IVH. (**B**) Beam walking scores; (**C**) BMS scores; (**D**) grip strength test of the hindlimb; (**E**) grip strength scores of all limbs; and (**F**) modified pole test. Values are shown as the mean ± S.D. N = 9 for each group, *p < 0.05.

The anatomical gross structure of ICH and IVH showed that the haematoma appeared in the internal capsule and lateral ventricle (Fig. 5A). In the grip strength test and modified pole test, the results showed that ICH mice behaved significantly worse than IVH mice from day 3 to day 28 (P < 0.05, Fig. 5D–F). However, in the BMS and beam walking test, the two models acquired no significantly different scores, except on the first day (P < 0.05, Fig. 5B,C). In the corner turn test, the mice in the ICH and IVH groups showed no significant differences (Supplementary Fig. 2).

To investigate if the tests could be influenced by non-motor factors, we did the 24-hour restraint experiments, which mainly cause the depression-like behaviour^31,32^. Mice which were subjected to 24-hour restrained showed reduced performance on the BMS and beam walking test. However, the restrained mice get the same scores as the mice of control group in grip strength and modified pole test (Supplementary Fig. 4).

### The strength test and modified pole test could distinguish the injury degree in ICH mice induced by different volumes of blood

**Figure 6:**
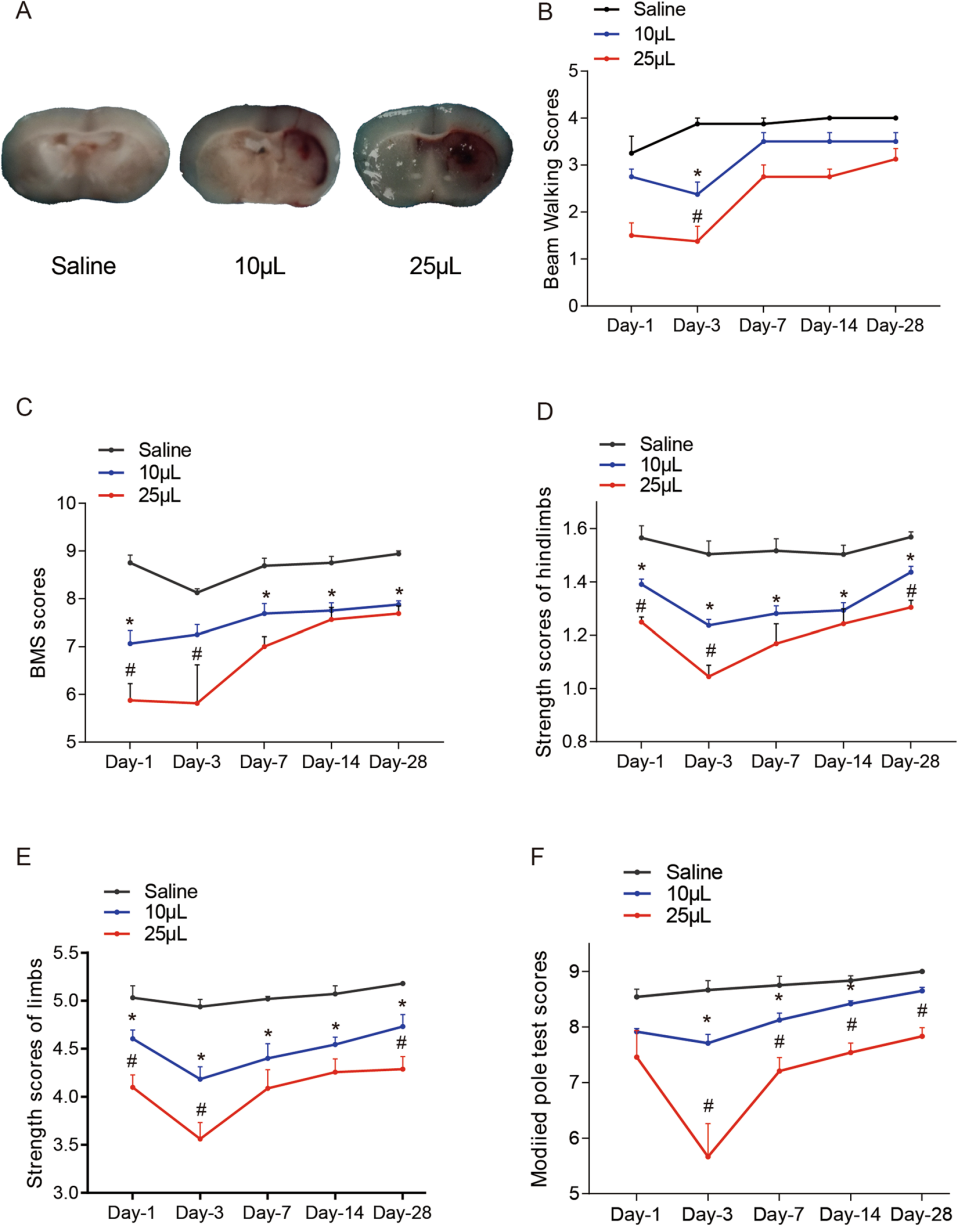
Mice injected with different volumes of blood were examined by behavioural tests. (**A**) The haematoma after ICH on day 1. (**B**–**F**) Behavioural tests; (**B**) beam walking scores; (**C**) BMS scores; (**D**) grip strength test of the hindlimb; (**E**) grip strength test of all limbs; and (**F**) modified pole test. Values are shown as the mean ± standard deviation. The 10 µl blood group was significantly different from the saline group is indicated by *p < 0.05. And the 10 µl blood group was significantly different from 25 µl blood group is indicated by ^#^p < 0.05. n = 6 for the saline and 10 µl blood groups, n = 9 for the 25 µl blood group.

The anatomical gross structure of different blood volume ICH models showed the haematoma in the internal capsule (Fig. 6A). In the BMS test and beam walking test, BMS scores showed that the neurological function of the 10 µl group was superior to that of the 25 µl blood group and worse than that of the saline group only on day 1 after ICH (P < 0.05, Fig. 6B,C). The grip strength test showed that the neurological function of the 10 µl group was superior to that of the 25 µl group and worse than that of the saline group on day 1, day 3 and day 28 after ICH (P < 0.05, Fig. 6D,E). The modified pole test showed that the neurological function of the 10 µl group was superior to that of the 25 µl blood group and worse than that of the saline group on day 3, day 7 and day 14 after ICH (P < 0.05, Fig. 6G). In the corner turn test, the mice in the ICH and IVH groups showed no significant differences (Supplementary Fig. 2).

## Discussion

The main results of this study are as follows. (1) The peak activation of microglia and astrocytes (i.e., the most severe time points of myelin loss and axonal swelling) occurred on the third day after intracerebral haemorrhage (ICH), which was also the time point when the worst grip strength and modified pole test results occurred. (2) The delay in motor-evoked potentials (MEPS) latency was the most severe on the third day after ICH. Grip strength test and modified pole test changes correlated with changes in MEPS latency. (3) The grip strength test and modified pole test could better detect the degree of injury between different volumes of the ICH model than the Corner turn, BMS test and beam walking test. (4) The grip strength test and modified pole test showed that the neurological function scores of ICH mice were significantly lower than that of intraventricular haemorrhage (IVH) mice, and were not affected by 24-hour restraint.

Basal ganglia brain parenchymal haemorrhage accounts for more than 80% of ICH^33^. The degree of white matter injury (WMI) detected on the second day after ICH can predict functional outcomes after one month^3^. The pathological process of white matter damage is mainly accompanied by damage and repair of the corticospinal tract (CST), which is closely related to motor function after cerebral haemorrhage^34,35^. Under normal conditions, intact myelin maintains CST transduction and axon function. During the onset of ICH, the reversible pathology of the CST involves demyelination and oedema^8,36,37^. Microglia and astrocyte responses can cause injury to the white matter surrounding the haematoma via inflammation in the acute phase from day 1 to day 7 after ICH^38,39^. Our study showed that there was a peak in the glial cell response and white matter damage around the haematoma on the third day after ICH, and the latency of MEPs was also the longest on day 3.

The grip strength test is a method used to accurately quantify the extent of muscle weakness and can eliminate weight change disturbances^40^. It was originally used to detect neuromuscular injury and skeletal muscle dystrophy^41,42^. It was later used to assess damage to the corticospinal tract after spinal cord injury^43,44^, but the grip test has not been used to assess WMI-induced motor function after ICH. Our results showed that changes in muscle strength were significantly associated with changes in the latency of MEPs. In addition, muscle grip was the worst on the third day, which was also the most severe time point for white matter lesions (Fig. 1). The pole test was first used to evaluate bradykinesia in MPTP-treated mice^45^. In this study, we modified the test to assess dyskinesia in unilateral limbs to focus on hemiplegia after basal ganglia haemorrhage. Our results showed that the modified pole test was well correlated with MEPS latency changes and was consistent with the degree of white matter pathological damage. These results indicate that the grip strength test and modified pole test can well reflect the damage caused by WMI after ICH.

The assessment of motor dysfunction can be divided into restricted motion assessments and free motion assessments based on the environment and requirements of behavioural testing of experimental animals. Restricted motion assessments are primarily affected by neuromuscular circuit injuries in the central nervous system, which are mainly caused by damage to brain motor neurons and the CST^46^, and include the Modified pole test and grip strength test. Free motion assessments, such as the Basso Mouse Scale (BMS), beam walking and corner turn are easily influenced not only by motor neurons and corticospinal tracts but also by pain, mood and learning^47^. Intraventricular haemorrhage is a type of brain injury that occurs in the non-white-matter area. Its main feature is pathological hydrocephalus, not white matter damage^48^. Here, the characteristics of these two different behavioural assessment methods were analysed in an ICH model and an IVH model. Our results showed that intraventricular haemorrhage did not cause MEPS delay on the third day. In addition, restricted motion assessment, rather than free motion assessment, can distinguish between the different motor deficits of ICH and IVH and were not affected by 24-hour restraint. Moreover, the assessment of restricted motor function not only can reflect white matter damage but can also distinguish the degree of damage in mice with different volumes of haematoma. In all, restricted motion behaviour tests are more sensitive than free motion assessments to dyskinesia induced by WMI after ICH. And these restricted motion assessments may be the potential behavioural test in the models of white matter damage in central nerves system diseases.

In summary, restricted motion assessments, such as the grip test and modified pole test, are a good way to distinguish the degree of motor impairment caused by white matter injury. These behavioural tests should be widely used when assessing motor deficits after ICH.

## Supplementary information

Supplementary information
