# Supplementary material for: Modified behavioural tests to detect white matter injury- induced motor deficits after intracerebral haemorrhage in mice
Source: Sci Rep. 2019 Nov 18;9:16958. doi: 10.1038/s41598-019-53263-6 (PMC6861313; doi:10.1038/s41598-019-53263-6)
Supplement: Supplementary file 1 — Supplementary information [file 41598_2019_53263_MOESM1_ESM.doc]

**Modified behavioural tests to detect white matter injury-induced motor deficits after intracerebral haemorrhage in mice**

Weixiang Chen^1,*^, Min Xia^1,*^, Chao Guo^1^, Zhengcai Jia^1^, Jie Wang^1^, Chengcheng Li^1^, Mingxi Li^1^, Xiaoqin Tang^1^, Rong Hu^1^, Yujie Chen^1^, Xin Liu^1^& Hua Feng^1^

^1^ Department of Neurosurgery, Southwest Hospital, Third Military Medical University (Army Medical University), 29 Gaotanyan Street, Shapingba District, Chongqing, 400038, China.

Corresponding author E-mail address: [Fenghua8888@vip.163.com](mailto:Fenghua8888@vip.163.com)

Supplementary methods:

Procedures of the 24-hour restraint. The mice were placed in a ventilated transparent plastic tube (diameter 3 cm, length 10 cm) and restraint for 24 hours from 10:00 am to 10:00 am of the next day. The mice were kept in the dark, passing through the air conditioning vents, accompanied by background noise. Holes in the head and side walls (0.5 cm in diameter) allow air to flow. Animals can move their heads and forelimbs, but their bodies and hindquarters cannot move or rotate. These animals have no food or water during the restraint. Once the restraint ended, mice were immediately put back to the cage and free to access food and water. Unconstrained mice (control group) stayed in the cage at home until the behavioral experiment began [^1^](#_ENREF_1).


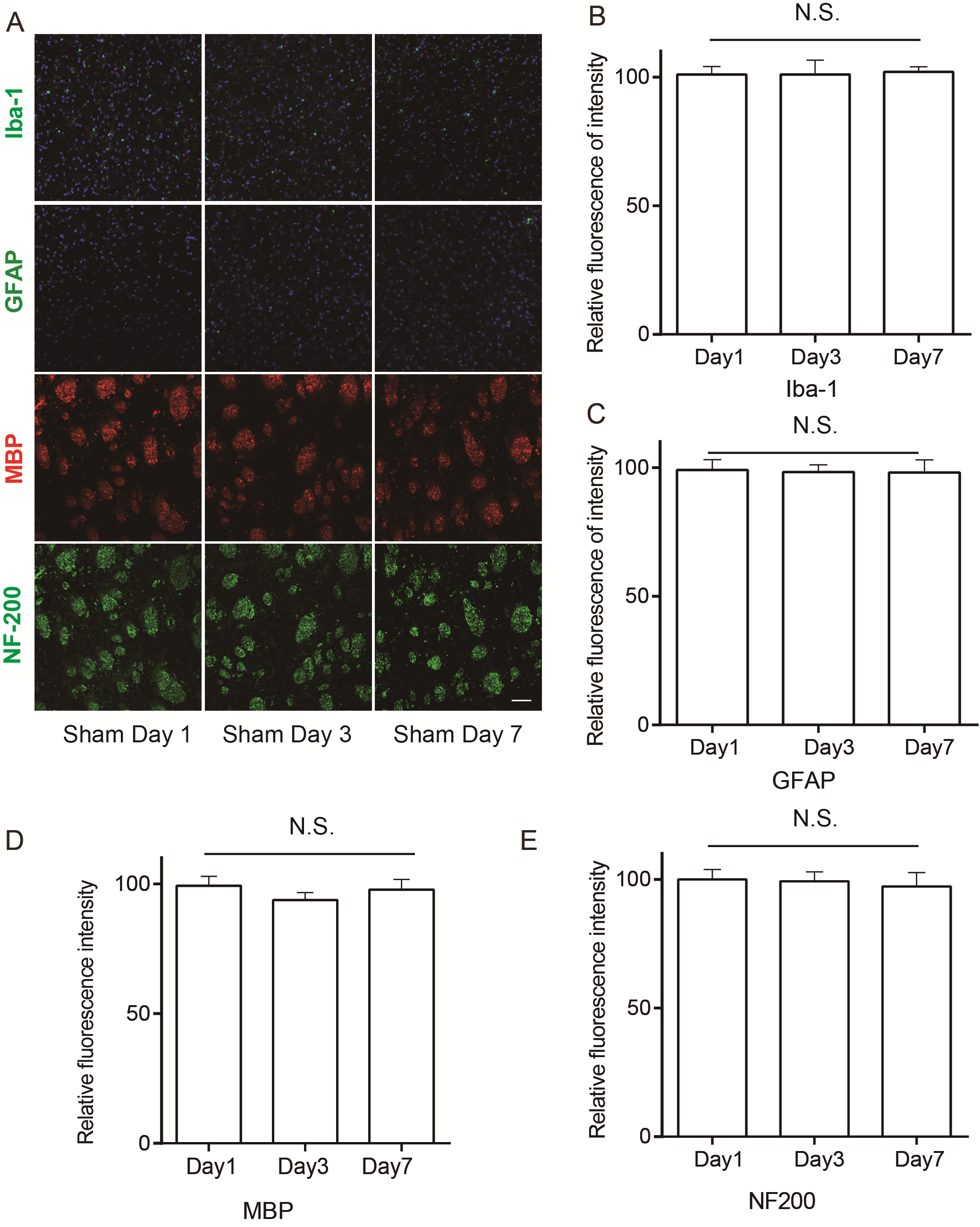


Supplementary figure 1: The glia cell and white matter shows no significant difference in the sham group. A). Iba1, GFAP-positive cells and MBP in brain sections were identified on days 1, 3 and 7 in the sham group. Immunoreactivity and of Iba1 (ionized calcium-binding adaptor molecule 1) and GFAP (glial fibrillary acidic protein; astrocyte marker) is shown in green, MBP (myelin basic protein) is shown in red, and NF-200 (neurofilament-200). (B-E) Box-plot graphs depicting the relative fluorescence intensity were showed in the figure. Values are shown as the mean ± S.D.. N=6 for each group. N.S. means no significant change.


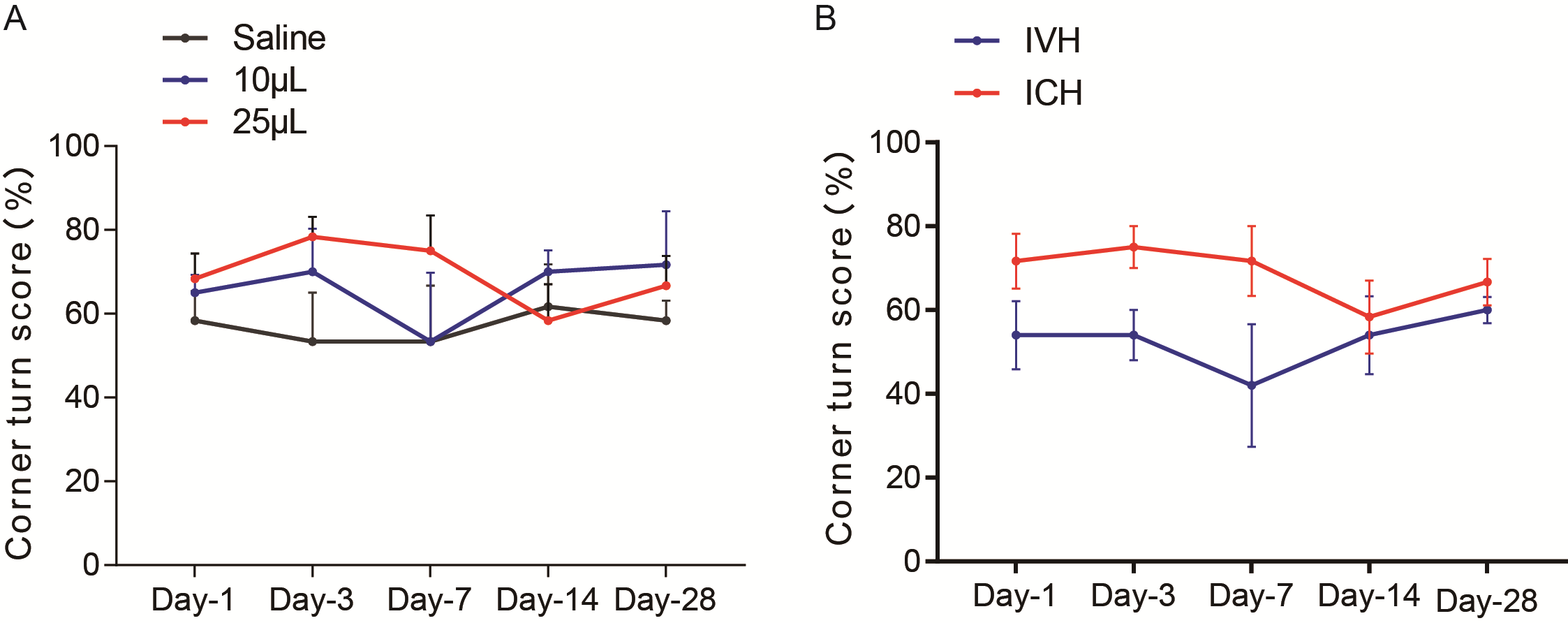


Supplementary Figure 2: There is no significant difference between 10 µl and 25µl blood group, as well as the ICH and ICH groups in the corner turn test. (A) Corner turn of mice after Saline and indicated volume of blood ICH model. (B) Corner turn of mice after ICH and IVH. Values are shown as the mean ±S.D.. N=7 for each group.


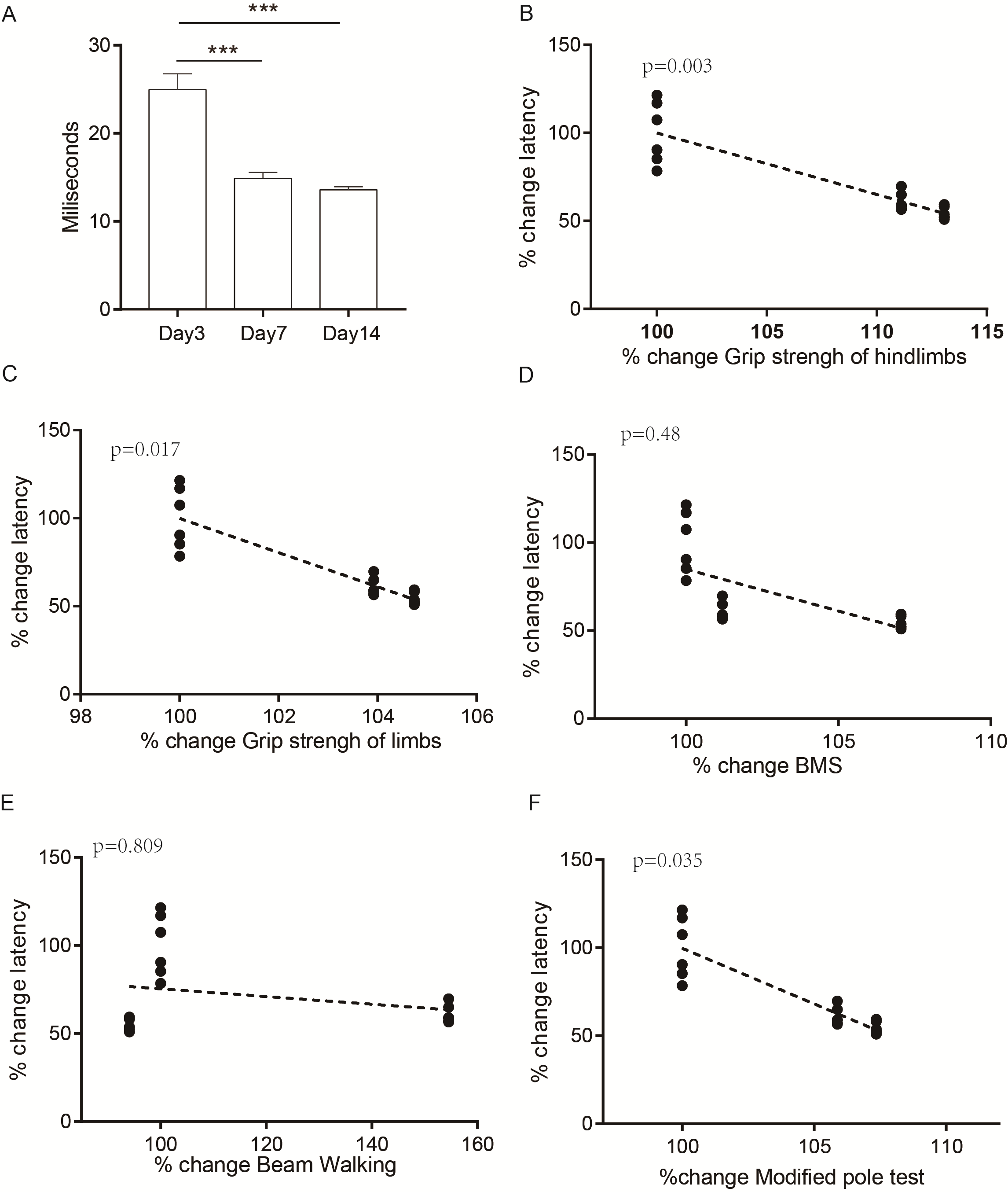


Supplementary Figure 3: The correlation between the percent change of different behavioural tests and the latency of MEPs. (A) The latency of motor-evoked potentials (MEPs) was quantified after ICH on day 3, day 7 and day 14. (B-F) The correlation between the percent change of different behavioural tests and the latency of MEPs were analysed from Day 3 to Day 14. n=6 mice per group.


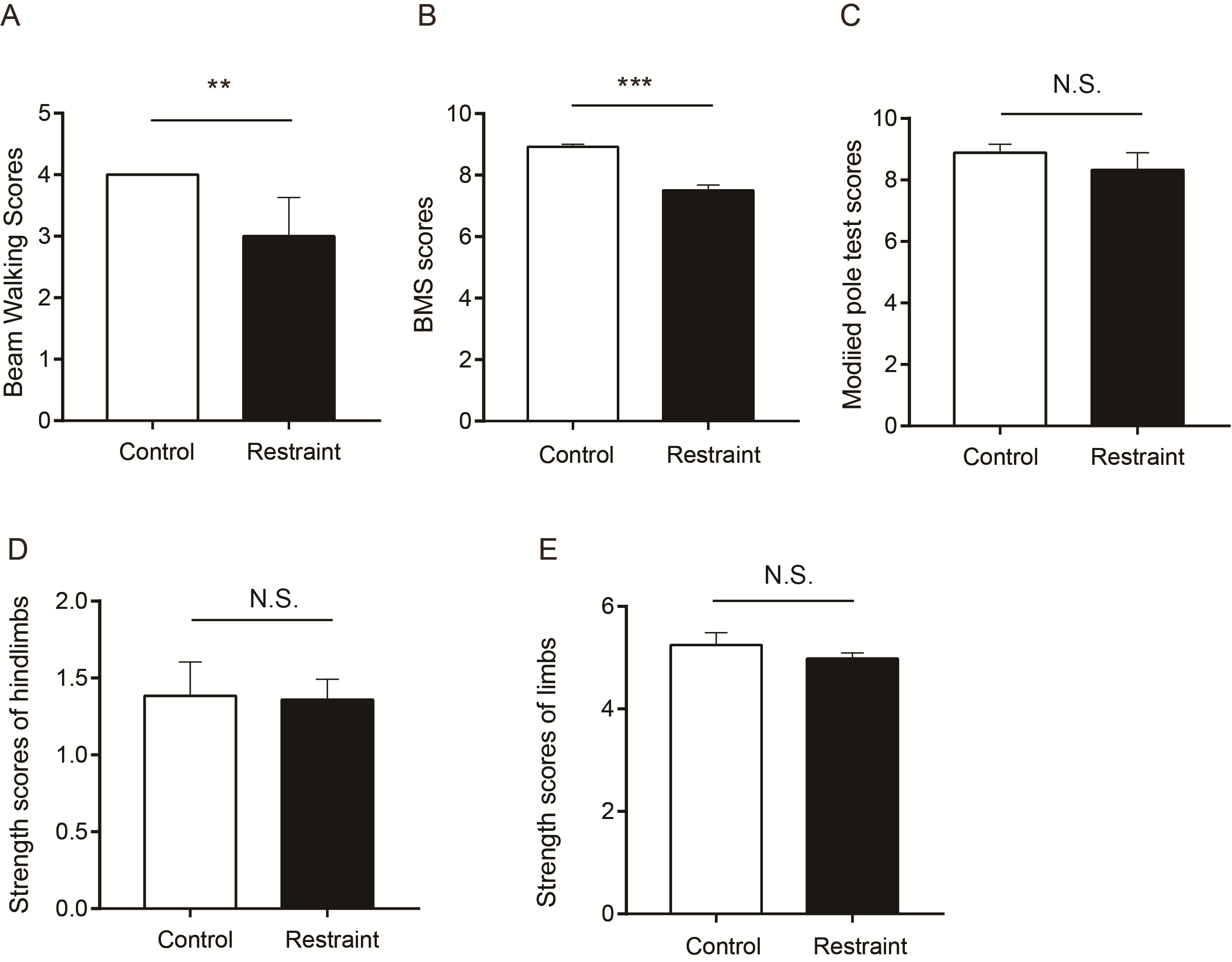


Supplementary Figure 4: Mice in restrained group got the lower scores than mice in the control group in BMS and beam walking test after 24-hour restraint experiment. (A-E) 24-hour restraint model was used and mice were scored by the behavioral assignments after 2 days. Values are shown as the mean ± S.D. N=6 for each group. **P<0.01, *** P<0.001 and N.S. means no significant change.

1 Chu, *X. et a*l. 24-hour-restraint stress induces long-term depressive-like phenotypes in mice*. Scientific repor*t**s** 6, 32935, doi:10.1038/srep32935 (2016).
